# Body image dissatisfaction and its relation to body mass index among female medical students in Sudan: across-sectional study 2020-2021

**DOI:** 10.1186/s12905-023-02748-8

**Published:** 2023-11-11

**Authors:** Balqees Abdeen Ahmed Mohamed, Malaz Hassan Dafaalla Idrees

**Affiliations:** Faculty of Medicine, University of Khartoum, ElQasr Ave, P.O. Box 103, Khartoum, Khartoum, Sudan

**Keywords:** Body image, Body mass index, Body image dissatisfaction, Eating disorders, University students, Sudan, Mental health in Sudan, Psychiatry in Sudan

## Abstract

**Background:**

Body image is mainly determined by biological, social, psychological and cultural factors thus it is a multifaceted vigorous construct. Body image is an essential aspect of girls' self-definition and individual identity. Excessive concern about body image and body image misconceptions leads to dissatisfaction, disturbed eating patterns, affecting the nutritional status and also leading to depression and anxiety disorder.

**Methods:**

This is a descriptive cross-sectional university-based study aiming to investigate body image dissatisfaction and its relation to BMI among female medical students at the University of Khartoum, faculty of medicine. The study was carried out between December 2020 and January 2021. Simple random sampling was applied and a two-sectioned questionnaire was used. The first part consisted of socio-demographic data and the second part contained questions to assess body image the data was. A total of 277 participants were enrolled in the study. Data was analyzed using SPSS version 20.

**Results:**

We enrolled 277 female medical students the majority of participants (53%) were considered of normal weight according to BMI, 7% considered obese, and 18% underweight. Large number of participants thought that they are not in the ideal weight according to their height (62%). (21% to 17%) of participants always feel pressure from people or society to get to a certain weight. With respect to attitude towards weight, (29%) of participants always wear clothes that don't reveal their body shape, (35%) of them always tend to wear clothes that hide their excess weight.

**Conclusions:**

The study concluded that participants who were overweight, obese or underweight have significant increase risk for poor body image perception with odd ratio of 39, 11, and 59 respectively. Thus early and proper interventions are necessary to circumvent the impact and future repercussion of body image distortion.

## Background

Body image is defined as one’s own discernment, thoughts, and emotions pertaining to one’s own body. It is the delineation of one’s body representation, including their mirror image, and it’s a reflection of the collective views of society, which is formed by society’s culture and norms. Body image perception is generated through body ideals, largely conveyed via the media, family, and peers [1]. Body image dissatisfaction occurs as a result of disparity between one’s perceived and ideal body image [2]. Body part discontentment, for instance, being dissatisfied with the size or shape of one’s body parts, including body shape, sex and sexual organs, also falls under the umbrella of body image distortion.

Self-appraisal regarding body image is generally shaped in late childhood and adolescence with personal worth strongly linked to beauty ideals, which is in turn linked to success, positive relationships, happiness and wealth [3].

Body image is shaped by societal expectations about what is deemed attractive and desirable in terms of body type [4]. In contrast to the western ideal of thinness, being fat is considered as a sign of femininity, fertility and motherhood [5]. In these societies, women’s primary role is motherhood, so being fat signifies their suitability for this role [6]. Historically, fatness was viewed as a sign of wealth, denoting excess resources in food-scarce settings and demonstrating that women were well taken care of by their husbands [7]. Thus, women may engage in fattening behaviors to conform to cultural standards.

A number of demographic factors have been associated with dissatisfaction with body image, such as being an adolescent or female, psychosocial factors, such as bullying exposure, lifestyle habits, such as inactivity and irregular eating patterns, and nutritional status, such as obesity [8].

A close relationship between BID and eating disorders is suggested in the scientific literature. Moreover, other studies attempt to examine the relationship between body image dissatisfaction and other psychological variables, including suicidality [9], sleep quality [10], unhealthy weight control behaviors [11] and depression [12, 13]. It is unclear whether concerns over physical appearance cause depression or whether depression increases a person's likelihood of developing BID [14].

Body Dissatisfaction and Distorted Body Image are common among university students [2] as well as a high prevalence of eating problems and insufficient physical activity [15]. The social milieu of young people alters when they go from high school to college. They make lifestyle decisions on their own without parental supervision, and the sudden increase in freedom may make it difficult for them to maintain a healthy lifestyle. At the same time, young adults need to cope with increased autonomy and academic pressure, making universities a fertile soil for mental health problems. Thus, it is very crucial to motivate university students to develop a healthy lifestyle [16].

This study was conducted to examine body image dissatisfaction among female students, particularly in the context of the higher prevalence of eating disorders in women [17]. By exploring these issues at a grassroots level, the research aims to identify potential risk factors and pave the way for preventive measures against eating disorders and related mental health issues. In addition to the societal pressures associated with being female, these students also experience escalating academic stress. Understanding these challenges is crucial to developing targeted interventions to support their overall well-being.

Changes in eating habits and body image are prevalent conditions, particularly in women and young people. Encouraging screening for these diseases is vital since they frequently go undetected, are becoming more common worldwide, and have already been linked to negative effects on both physical and mental health.

However, studies exploring body dissatisfaction are in abundance in the literature of western countries and in some non-western countries this issue is still under-reported in Sudan. In this light, this study aims to provide evidence upon which the necessary interventions can be conducted.

## Methods

### Study design

This is a descriptive cross-sectional university-based study aiming to investigate body image dissatisfaction and its relation to BMI among female medical students at the University of Khartoum, faculty of medicine. The study was carried out between December 2020 and January 2021.

### Study setting

Faculty of medicine, University of Khartoum, located in northern Khartoum. The faculty of medicine was founded in 1924 as Kitchener School of Medicine. It is one of the oldest medical schools in the Arab world, has more than 800 released research articles, 100 batches and 48 departments. It was also the first medical school in Sudan to be accredited by the world federation of medical education. The University of Khartoum is generally recognized as a top university and a high-ranked academic institution in Sudan and Africa.

### Study population

In terms of the inclusion criteria, all female medical students studying at the University of Khartoum, Faculty of medicine during the academic year 2020–2021 were included in the study. Only males were excluded from the study.

### Sample size and sampling

According to the faculty administration, a total of 2343 students were enrolled at the Faculty of Medicine, University of Khartoum, in the years 2020–2021, approximately two-thirds of whom (*N* = 1562) are female. A total of 277 participants were enrolled in the study using non-probability convenience sampling.

The sample Calculated using the following formula: n = (z)2 p (1 – p)/d2

n = minimum sample size

z = the normal standard deviate, (z = 1.645) for a confidence level of 90% p = proportion (estimated as 0.5) d = the level of precision (0.05)

According to the previously mentioned formula, the sample size was estimated to be a minimum of 270 participants.

### Data collection methods and tools

The study was conducted during the period of COVID-19 lockdown therefore an online self-administered questionnaire was sent to the participants on WhatsApp and Telegram. Before engaging in the questionnaire, participants were asked to give informed consent for participation by answering the question ―I agree to participate in this study.

The questionnaire used was structured from previous literature [18–20] and a pilot study of 15 samples was carried out to test for reliability with a Cronbach’s alpha of 0.846.

The questionnaire consisted of two sections. The first section contained demographic data like age, academic year, definite or estimated height and weight to calculate body mass index.

Body mass index was calculated using the following formula: Body mass index = (weight in kilograms) / (height^2^ in meters), according to the obtained definite or estimated weight and height.

The second part of the questionnaire was comprised of 24 questions to assess body image, 5 questions inquiring about the psychological domain, 6 questions for the social domain, 6 questions to explore the overall body perception, 2 questions to inquire about body part perception and 4 questions to study the attitude towards weight. A question asking participants whether they think that their weight is appropriate for their height was also added. The responses were recorded on a 7 point Likert scale from 0 to 6 (0 = never, 1 = rarely, 2 = occasionally, 3 = sometimes, 4 = often, 5 = usually, 6 = always).

### Data collection, management and analysis

Questionnaires were refined and managed carefully; completeness was checked before data entry. Data was analyzed using SPSS version 20. And presented as frequencies and percentage. An independent sample binary logistic model was used to predict the risk of poor body image perception with a p value less than 0.05 considered significant.

## Results

**Fig. 1:**
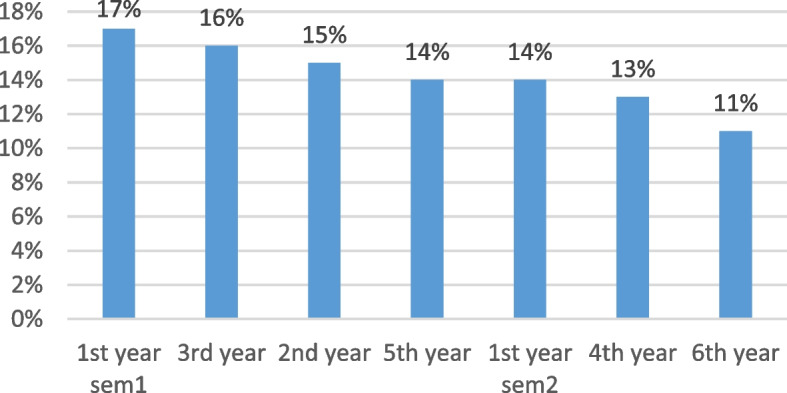
Distribution of participants by academic year. Demonstrates the distribution of participants by academic year. Ranging between 11% (6^th^ year) and 17% (1.^st^ year semester)

**Fig. 2:**
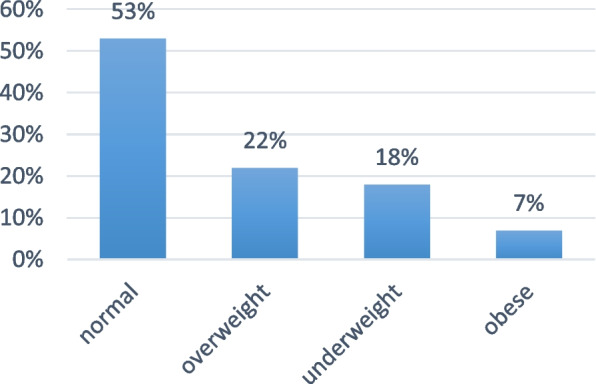
Weight status of the participants. Demonstrates the weight status of the participants according to body mass index. With the majority of them having normal weight (53%)

We enrolled 277 female medical students from different academic years and ages, the mean weight was considered normal with majority of participants according to BMI 53%while 7% considered obese, 22% overweight and 18% underweight (Figs. 1 and 2).

Regarding the psychological domain, a significant percentage of the participants always feel embarrassed about their physical appearance and have thought of dissatisfaction with their bodies (22%and 26% respectively). An appreciable percentage of them never shut down when they feel bad about their body shape or weight irrespective of their actual weight (30%). An appreciable percentage of the participants (22%) stated they are always sensitive to people commenting on my weight in general (Table 1).

**Table 1:** Psychological domain

	Always	Usually	Often	Sometime**s**	Occasionally	Rarely	Never
I sometimes get upset I don't look good as others	20%	14%	9%	14%	11%	15%	18%
I feel embarrassed about my physical appearance	22%	11%	11%	16%	8%	13%	19%
I am critical of my body/I have thoughts of dissatisfaction with my body	26%	9%	11%	9%	8%	15%	23%
I shut down when I feel bad about my body shape or weight (thin/normal weight but not desired shape or overweight)	16%	12%	6%	10%	10%	15%	30%
When I start thinking about my body shape or weight it's hard to stop	17%	10%	9%	9%	5%	16%	33%

Shows that a significant percentage of the participants always feel embarrassed about their physical appearance and have thought of dissatisfaction with their bodies (22%and 26% respectively)

On the social domain, (55%) of the respondents are always sensitive that others found them skinny. (22%) of respondents are always sensitive to people commenting on their weight. (21% to 17%) of participants always feel pressure from people or society to get to a certain weight. (29%) never feel physically intimidated by others (Table 2).

**Table 2:** Social domain

	Always	Usually	Often	Sometimes	Occasionally	Rarely	Never
I am sensitive that others find me skinny	55%	8%	4%	2%	6%	16%	8%
I am sensitive to people commenting on my weight in general	22%	14%	8%	7%	9%	16%	25%
I feel that I am physically intimidated by others	19%	10%	9%	9%	8%	16%	29%
The first thing that people notice about me is that I am overweight	25%	15%	5%	20%	5%	0%	30%
People (Family, Friends…etc.) Pressure me to get to a certain weight	21%	12%	11%	10%	11%	8%	27%
I feel extreme pressure by society due to my weight	17%	10%	12%	5%	11%	18%	28%

Shows the participants’ perceived societal feedback regarding their weight

Regarding the overall body perception and body part perception, (40%) of participants always feel that they are a bit overweight, on another hand (73%) of them feel that they are thin and want to gain weight. (40%) thought that they have normal weight but not the desirable body shape, (23%) feel less physically attractive (Table 3) (40%) of the participants don ‘t like their protruding stomach (Table 4).

**Table 3:** Overall body perception

	Always	Usually	Often	Sometimes	Occasionally	Rarely	Never
I feel that I am a bit overweight	40%	10%	0%	20%	10%	5%	15%
I feel that my weight is not evenly distributed throughout my body	27%	10%	9%	9%	8%	12%	25%
I feel that to control my life I have to control my weight	21%	9%	12%	8%	12%	13%	26%
I feel less physically attractive	23%	11%	12%	9%	10%	17%	17%
I feel that I'm thin I want to gain weight in certain areas	73%	12%	4%	4%	4%	2%	0%
I have normal weight but not the body shape that I desire	15%	7%	9%	5%	9%	16%	40%

Shows the participants’ feelings and thoughts with regard to their weight and body shape

**Table 4:** Body part perception

	Always	Usually	Often	Sometimes	Occasionally	Rarely	Never
I don't like the size of my Arms/shoulders	20%	8%	9%	6%	9%	12%	37%
I don't like my protruding stomach	40%	0%	10%	10%	15%	10%	15%

Shows the participants feelings and thoughts with regard to their body parts 40% of the participants do not like their protruding stomach always

With respect to attitude towards weight, (29%) of participants always wear clothes that don't reveal their body shape, (35%) of them always tend to wear clothes that hide their excess weight (Table 5).

**Table 5:** Attitude toward weight

	always	Usually	Often	Sometimes	Occasionally	Rarely	Never
I wear clothes that won't reveal my body shape	29%	12%	8%	11%	10%	12%	17%
I tend to wear clothes to hide my excess weight	35%	10%	5%	15%	5%	0%	30%
I diet in order to control my weight	10%	8%	8%	10%	12%	19%	34%
I would love to have a narrow waist	34%	13%	12%	8%	8%	11%	14%

Demonstrates how the participants behave with regard to their weight. An appreciable percentage of the participants reported that they wear clothes that doesn’t reveal their body shape (29%)

**Fig. 3:**
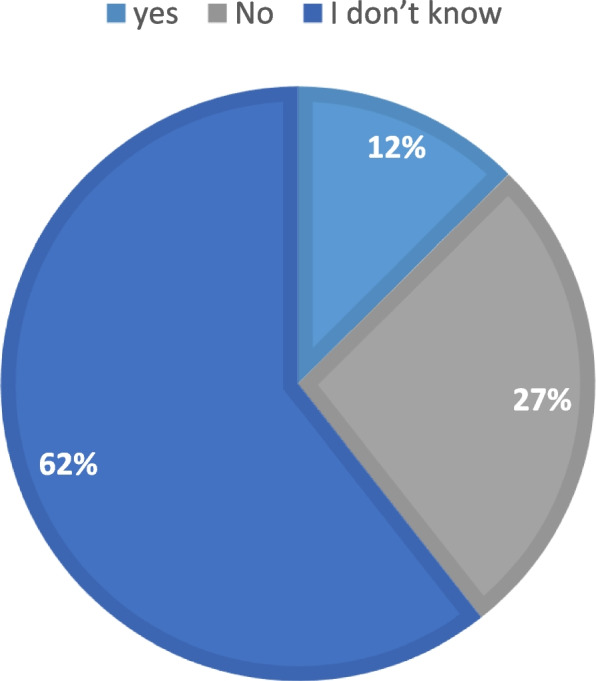
Ideal weight according to height. Demonstrates whether the participants are in the ideal body mass index. Majority of the participants (62%) do not think that they are in the ideal weight according to their height

Majority of participants thought that they are not in ideal weight according to their height (62%) (Fig. 3).

Participants who were overweight, obese or underweight have significant increase risk for poor body image perception with odd ratio of 39, 11, and 59 respectively (Table 6).

**Table 6:** Risk for poor body image perception

Status	Odd ratio	*P* value	95% CI for odd ratio	
			**Lower**	Upper
Overweight	39.382	.000	9.219	168.237
Underweight	59.418	.000	7.939	444.680
Obese	11.745	.000	2.606	52.940

Shows that participants who were overweight, obese or underweight have significant increase risk for poor body image perception with odd ratio of 39, 11, and 59 respectively

## Discussion

This study aims to explore body image dissatisfaction and its relation to body mass index among female medical students in Sudan. Although body image dissatisfaction has been widely studied in the literature, it’s relatively under-reported in Sudan. This is a pioneer study that provides evidence on this subject.

Two hundred seventy-seven female medical students were enrolled from different academic years and different ages. The majority of the respondents mean weight was found to be normal according BMI (53%),22% were overweight, 7% were considered obese and (18%) were underweight.

In this study, it was found that participants who were overweight, obese or underweight had a significantly increased risk for poor body image perception with an odd ratio of 39, 11, and 59 respectively. A systemic review and meta-analysis in which 17 articles were used aimed at systematically investigating the degree of body dissatisfaction in individuals with obesity compared to individuals with normal weight, as well as exploring gender variations in body dissatisfaction. This meta-analysis has established that the group with obesity was significantly affected by body dissatisfaction in comparison to the normal weight group. It also revealed that the difference in body dissatisfaction between women with obesity and normal weight is significantly higher than in men [14]. In a study aiming to examine body image perceptions and body image dissatisfaction and their relationship with body mass index (BMI) among medical students in Oman, it was noted that students with obesity were four times more likely to develop body image distortion compared to underweight students [2].

This might be ascribed to the social stigma associated with “being fat” [21].

Furthermore, slightly more than a half of the respondents are always sensitive that others found them skinny (55%) and 73% of them feel that they are thin and want to gain weight in certain areas. This might be influenced by the societal norms in Africa that perceive fatness as a sign of wealth and happiness. This ideal still runs deep within African societies despite westernization and modernization. This is reinforced by the findings of a systemic review in which 73 articles from 21 countries were conducted to demonstrate evidence on body size preferences for females living in Africa and the factors influencing them. With a notable preference for normal or overweight body sizes. Preferences for larger body sizes are influenced by psycho-social factors such as avoiding HIV stigma and socio-cultural factors such as a spouse’s preference, social standing and cultural norms [4].

29% of participants always wear clothes that don't reveal their body shape, 35% of participants always tend to wear clothes that hide their excess weight. Early in childhood, beginning with the process of getting to know one's own body, attitudes toward one's body are created. Then, through comparisons with those around us, their views of us, and their attitudes toward us, these attitudes are developed and internalized. Compared to men, girls and women's perception of their bodies form a substantially larger part of who they are, and this has a significant impact on their total self-esteem [22].

22% of respondents stated that they’re always sensitive to people commenting on their weight. (21% to 17%) of participants always feel pressure from people or society to get to a certain weight. (29%) never feel physically intimidated by others. It appears that the caliber of social feedback or the incorrect interpretation of such feedback has an effect on our psychological well-being. In fact, it is well established that body image distortion gives rise to eating disorders and depression [23]. Fasting, very strict diets, diuretics, and diet pills were all documented as harmful weight-controlling behaviors, particularly among girls [24].

Participants exhibited several concerns pertaining to their body shape or perceived attractiveness: 40% thought that they had a normal weight but not the desirable body shape; 23% felt less physically attractive; and 62% thought they were not of ideal weight according to their height. 40% of the participants don ‘t like their protruding stomachs. A study conducted among female undergraduate students in Delhi University's North Campus colleges revealed that 30.6% of participants had body shape concerns, with 7.3% showing moderate to marked concerns. These concerns were notably linked to nutritional status and media influences, indicating the significant impact of both personal health and societal pressures on the participants' body image perceptions [25]. In addressing body image dissatisfaction and related challenges, three distinct coping strategies have been identified: avoidance (ignoring bad thoughts about your body), appearance fixing (trying to hide or fix things you don't like about your body), and acceptance coping (focusing on positive aspects and self-care) [26].

An appreciable percentage of them never shut down when they feel bad about their body shape or weight, irrespective of their actual weight (30%). This resilience may be attributed to effective coping strategies. A study among first-year college students examined how young women handle body image concerns. The research identified different coping methods and evaluated how effective they were perceived to be. Most participants mentioned exercise as their go-to strategy, followed by healthy eating, altering appearance, confiding in friends or family, turning to religion or spirituality, spending time alone, engaging in activities, and practicing self-acceptance [27].

Moreover, the study had several limitations. Firstly, the study included females from only one college. Thus, these findings may not be generalizable to all female medical students in Sudan. Secondly, as the study was conducted during the period of COVID-19 lockdown, access to the participants was quite restricted, thus an online self-administered questionnaire was used instead of using an in-depth psychiatric interview, which largely depended on the participants’ honesty, introspective ability and interpretation of questions subjecting the outcomes to bias. Finally, self-reported definite or estimated weight and height was used to calculate body mass index, which may result in bias due to under or overestimation of values.

## Conclusions

The study concluded that Participants who were overweight, obese or underweight have a significantly increased risk for poor body image perception with odd ratio of 39, 11, and 59 respectively. A healthy body image doesn’t only affect students’ self-esteem and satisfaction but also their overall mental health. poor body image is associated with reduced quality of life and performance necessitating proper interventions. Early screening, awareness campaigns amongst schools could facilitate the spread of information regarding correct awareness of healthy body image as well as integration into the medical school curriculum.

## Data Availability

The datasets used and analyzed during the current study are available from the corresponding author upon reasonable request.
